# Mortality in cardiogenic shock patients receiving mechanical circulatory support: a network meta-analysis

**DOI:** 10.1186/s12872-022-02493-0

**Published:** 2022-02-13

**Authors:** Qun Zhang, Yu Han, Shukun Sun, Chuanxin Zhang, Han Liu, Bailu Wang, Shujian Wei

**Affiliations:** 1 Department of Emergency and Chest Pain Center, Qilu Hospital, Cheeloo College of Medicine, Shandong University, NO. 107, Jinan, 250012 Shandong China; 2 Clinical Research Center for Emergency and Critical Care Medicine of Shandong Province, Institute of Emergency and Critical Care Medicine of Shandong University, Qilu Hospital, Cheeloo College of Medicine, Shandong University, Jinan, 250012 Shandong China; 3 Key Laboratory of Emergency and Critical Care Medicine of Shandong Province, Key Laboratory of Cardiopulmonary-Cerebral Resuscitation Research of Shandong Province, Qilu Hospital, Cheeloo College of Medicine, Shandong University, Jinan, 250012 Shandong China; 4 The Key Laboratory of Cardiovascular Remodeling and Function Research, Chinese Ministry of Education, Chinese Ministry of Health and Chinese Academy of Medical Sciences; The State and Shandong Province Joint Key Laboratory of Translational Cardiovascular Medicine, Qilu Hospital, Cheeloo College of Medicine, Shandong University, Jinan, 250012 Shandong China; 5 Clinical Trial Center, Qilu Hospital, Cheeloo College of Medicine, Shandong University, Jinan, 250012 Shandong China

**Keywords:** Cardiogenic shock, Mechanical circulatory support, Venoarterial extracorporeal membrane oxygenation, Intra-aortic balloon pump, Impella, Tandem heart

## Abstract

**Objective:**

Mechanical circulatory support (MCS) devices are widely used for cardiogenic shock (CS). This network meta-analysis aims to evaluate which MCS strategy offers advantages.

**Methods:**

A systemic search of PubMed, EMBASE, and the Cochrane Central Register of Controlled Trials was performed. Studies included double-blind, randomized controlled, and observational trials, with 30-day follow-ups. Paired independent researchers conducted the screening, data extraction, quality assessment, and consistency and heterogeneity assessment.

**Results:**

We included 39 studies (1 report). No significant difference in 30-day mortality was noted between venoarterial extracorporeal membrane oxygenation (VA-ECMO) and VA-ECMO plus Impella, Impella, and medical therapy. According to the surface under the cumulative ranking curve, the optimal ranking of the interventions was surgical venting plus VA-ECMO, medical therapy, VA-ECMO plus Impella, intra-aortic balloon pump (IABP), Impella, Tandem Heart, VA-ECMO, and Impella plus IABP. Regarding in-hospital mortality and 30-day mortality, the forest plot showed low heterogeneity. The results of the node-splitting approach showed that direct and indirect comparisons had a relatively high consistency.

**Conclusions:**

IABP more effectively reduce the incidence of 30-day mortality compared with VA-ECMO and Impella for the treatment of CS.

**Supplementary Information:**

The online version contains supplementary material available at 10.1186/s12872-022-02493-0.

## Introduction

Cardiogenic shock (CS) is a state of low cardiac output and hypoperfusion that is highly associated with organ damage [1]. The progress made in the field of mechanical circulatory support (MCS) has led to considerable changes in the management and treatment of CS; however, CS remains associated with a certain degree of mortality [2]. In clinical practice, venoarterial extracorporeal membrane oxygenation (VA-ECMO) has been frequently used to treat CS caused by different aetiologies such as postcardiotomy shock, acute myocardial infarction (AMI), end-stage heart failure, and acute myocarditis [1, 3–7].

CS continues to be associated with high rates of mortality and morbidity, causing a therapeutic challenge for clinicians [1, 8–10]. Although the mortality of CS patients may decrease over time, the short-term mortality rate remains 35–40% [11–13]. The main cause of CS is myocardial infarction (MI) [11]. Nevertheless, even after active treatment, there is a high mortality rate, so it is particularly important to reduce short-term mortality [11, 14]. MCS has achieved considerable advances in the treatment of CS and MCS has a theoretical basis for the treatment of CS. Moreover, this treatment has been accepted by clinicians. Therefore, the purpose of this study was to evaluate the in-hospital mortality and 30-day mortality of CS patients who underwent MCS treatment, to provide the best intervention strategy for clinicians.

## Methods

This network meta-analysis (NMA) complies with the Preferred Reporting Items of Systematic Reviews and Meta-Analyses (PRISMA) guidelines [15]. All aspects involved in this study were independently conducted by at least two researchers.

### Inclusion criteria

Study types: Studies included double-blind, randomized controlled, and observational trials, with 30-day follow-ups.

Participants: Patients included adults and children diagnosed with CS. CS diagnostic criteria have been debated over the years. Clinicians established the presence of CS by combining evidence of end-organ dysfunction and abnormal haemodynamic parameters. Most patients were diagnosed based on some combination of the following diagnostic criteria: (I) severe hypotension with systolic blood pressure (BP) < 80–90 mmHg for at least 30 min, the mean BP decreases by 30 mmHg or more from baseline, and vasoactive medications are needed to maintain the systolic BP above 90 mmHg in spite of sufficient fluid resuscitation; (II) elevated biventricular filling pressures with pulmonary capillary wedge pressure (PCWP) exceeding 15 mmHg and central venous pressure above 10 mmHg; (III) significantly reduced cardiac index (< 1.8 L/min/m^2^ or < 2.2 L/min/m^2^ with haemodynamic support); (IV) low mixed venous blood oxygen saturation signalling increased peripheral oxygen extraction due to hypoperfusion [13, 16].

Interventions: The interventions for CS included Tandem Heart(Cardiac Assist, Pittsburgh, PA, USA)plus Impella, medical therapy, VA-ECMO plus intra-aortic balloon pump(IABP), Tandem Heart, IABP, Impella, VA-ECMO, VA-ECMO plus Impella, Impella plus IABP, and Surgical Venting plus VA-ECMO.

### Retrieval strategy

To identify relevant clinical trials, we searched PubMed, EMBASE, and the Cochrane Central Register of Controlled Trials. To expand the number of included studies, the search terms “cardiogenic shock” and “mechanical circulatory support” were used. The researchers screened the literature according to the inclusion criteria of this study. After two researchers determined that an article satisfied the preliminary inclusion criteria by reading the title and abstract, the researchers proceeded to read the full text independently to finally determine whether the article met the inclusion criteria. When differences were noted, the two researchers discussed the inclusion qualification of the article until they reached an agreement. If no agreement could be reached, a third researcher acted as an arbitrator to determine whether the article met the inclusion criteria. The reference lists of all included studies were also screened to examine relevant articles and discover other related published and unpublished research. To minimize publication bias, clinical trial registries (ClinicalTrials.gov[http://clinicaltrials.gov/]) were searched. Any discrepancies in the selected papers were resolved by consensus.

### Data extraction and clinical outcome

A data extraction form was used by two pairs of reviewers to extract data independently and duplicate them. The name of the project or the last name of the first author, the time of publication, study design, setting, aetiology of CS, and interventions (VA-ECMO plus IABP, IABP, VA-ECMO, medical therapy, VA-ECMO plus Impella, percutaneous left ventricular (LV) assist devices (PLVADs)) were extracted. We considered “no MCS used” described by the study authors as “medical therapy” and extracted quantitative data from the studies. The number of patients who died in the hospital, those who died within 30 days, and the total number of patients receiving treatment were extracted. The primary outcomes were in-hospital mortality and 30-day mortality.

### Meta-analysis methods and quality assessment

Using fixed-effects models [17], a Bayesian NMA was conducted using netmeta [18]. The NMA was used to estimate the relative effectiveness of all interventions for the primary outcomes by using a fixed-effects model combined with direct and indirect evidence. The model assumes that the between-study heterogeneity parameters and frequency theory methods of the whole network are common. We conducted NMA using the package netmeta in R software (Version 4.0.3, http://www.r-project.org/). The design-by-treatment test (global) and the node-splitting approach were used to perform a statistical evaluation of consistency. The Bayesian analyses estimated rank probabilities. The probability of each treatment obtaining each possible rank is shown by their relative effects. Odds ratios (ORs) and 95% confidence intervals (CIs)were used to evaluate the efficacy of various MCS equipment for adverse clinical events. To visualize heterogeneity, prediction intervals were used in the forest plots for the primary outcomes. We assessed network heterogeneity by the I^2^ statistic. I^2^ > 50% indicated higher heterogeneity. The fixed-effects model was used first. When I^2^ was > 50%, a random-effects model was used for statistical analysis. Subgroup analysis was performed to explore the causes of heterogeneity. Sensitivity analysis was performed by omitting each study to evaluate the reliability and stability of all studies. The methodological quality of the included articles was assessed according to the Cochrane Risk of Bias criteria [19]. Cumulative ranking plots and the surface under the cumulative ranking (SUCRA) were used to rank the advantages and disadvantages of interventions. The quality of retrospective and randomized controlled trials was evaluated by the Newcastle–Ottawa Scale and the Jadad score, respectively. Funnel plots were used to assess potential bias. Finally, the results were incorporated into the CINeMA application to assess the credibility of the results from each NMA [20]. CINeMA grades the confidence for the results of each intervention comparison as high, moderate, low, or very low. The statistical analyses in this NMA were performed using a combination of R software (Version 4.0.3, http://www.r-project.org/), STATA statistical software (version 16; StataCorp, College Station, Texas, USA), and Review Manager software (Version 5.3; Copenhagen; The Nordic Cochrane Center, The Cochrane Collaboration, 2014).

## Results

### Study characteristics

**Fig. 1:**
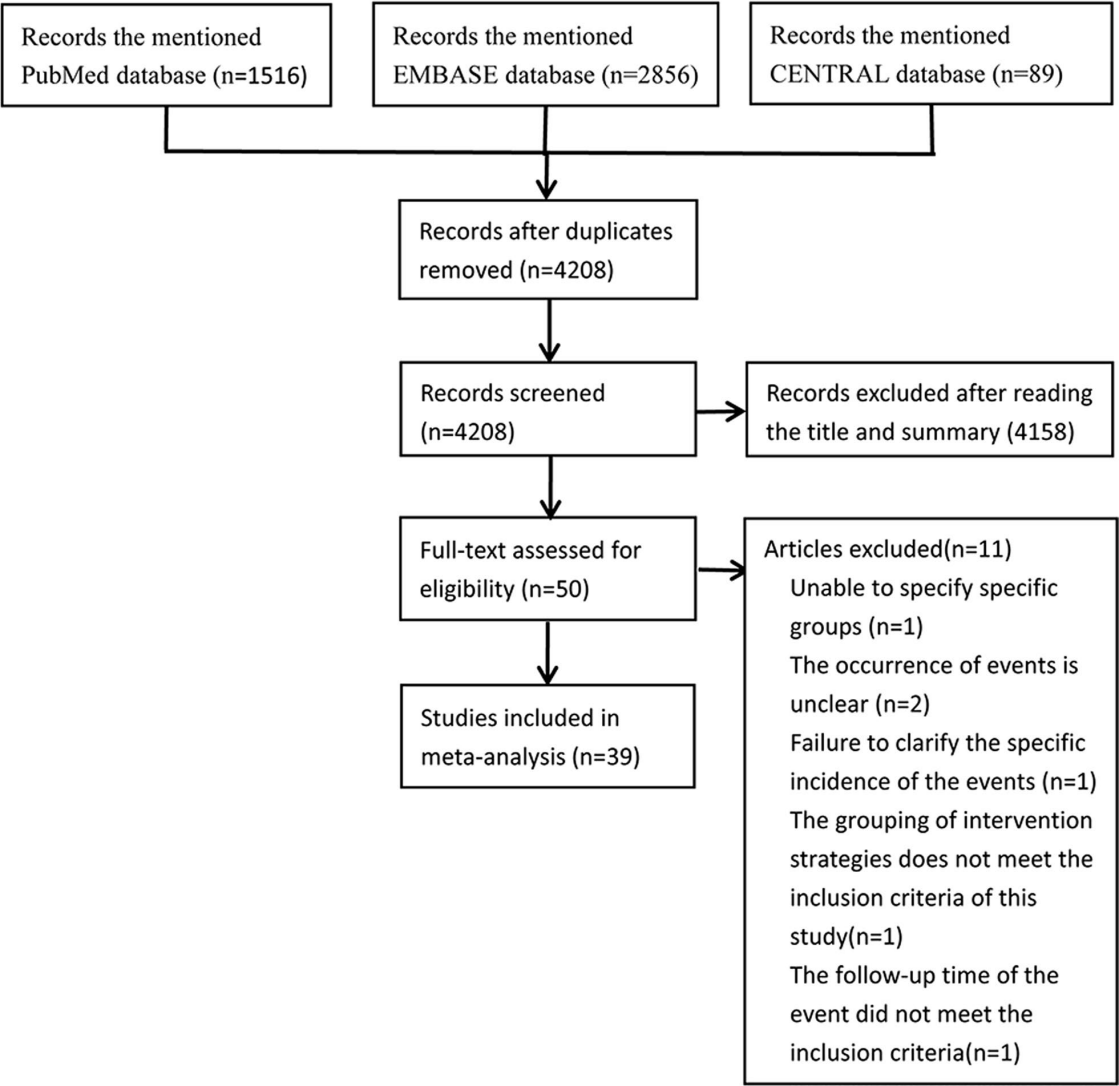
The flow chart of literature retrieval and reasons for article exclusion

A total of 4461 articles were retrieved by searching relevant online databases. Of these,253 articles were eliminated due to duplication. By retrieving the references of previous meta-analyses, 26 additional articles met the inclusion criteria. After reading the title and abstract, 4158 articles were excluded and 50 were identified. Thereafter, 11 articles were removed after reading the full text. The flow chart of literature retrieval and reasons for article exclusion are shown in Fig. 1. Finally, we included 39 studies (including 1 report) in this NMA [11, 21–57]. The quality assessment of studies that met the inclusion criteria is shown in Additional file 1: Figure S1.

A total of 10,985 patients were included in this meta-analysis. 9 double-blind, randomized controlled trials and 30 observational trials were identified. The interventions included VA-ECMO plus IABP, VA-ECMO, IABP, medical therapy, VA-ECMO plus Impella, and PLVADs (Impella, Tandem Heart). The clinical safety of VA ECMO plus IABP and IABP, PLVADs with IABP, VA-ECMO plus IABP with VA-ECMO, PLVAD with medical therapy, IABP with medical therapy, Impella plus VA-ECMO with Impella, VA-ECMO plus Impella with VA-ECMO, and VA-ECMO with Impella was compared in 3, 8, 4, 1, 11, 1, 6, and 5 articles, respectively. The characteristics of all studies that met the inclusion criteria are summarized in Table 1. The study designs of all randomized controlled trials were of high quality according to the Cochrane Risk of Bias criteria.

**Table 1:** The characteristics of all studies

Study	Year	No. of participants	Study design	Setting	Etiology of CS	Quality assessment
ECMO plus IABP vs. IABP						
Perazzolo Marra et al.	2013	35	Obs	Europe	AMI	5
Tsao et al.	2012	58	Obs	Asia	AMI	7
Sheu et al.	2010	219	Obs	Asia	STEMI	9
PLVADs vs IABP						
Seyfarth et al. (ISAR-SHOCK)	2008	26	RCT	Europe	AMI	7
Schrage et al	2018	352	Obs	Europe	AMI	9
Bochaton et al	2019	13	RCT	Europe	AMI	4
Dagmar et al. (IMPRESS trial)	2016	48	RCT	Europe	AMI	7
Shah et al	2012	27	Obs	United States	STEMI or UA/NSTEMI	6
Thiele et al	2005	41	RCT	Europe	AMI	7
Manzo-Silberman et al.	2013	78	Obs	Europe	ACS	9
Burkhoff et al.	2006	33	RCT	United States, Europe	AMI (70%)	5
Schwartz et al.	2012	76	Obs	United States	STEMI (68%)	7
ECMO plus IABP vs. ECMO						
Park et al.	2014	96	Obs	Asia	AMI	8
Chung et al.	2011	20	Obs	Asia	AMI	5
Aoyama et al.	2014	38	Obs	Asia	AMI, INCA (2 pts, OHCA 7 pts)	6
PLVAD vs. medical therapy						
Feistritzer et al.	2020	1024	RCT	Europe	AMI	7
IABP vs medical therapy						
Sanborn et al. (SHOCK Registry)	2000	383	Obs	United States, Canada, Europe, New Zealand	AMI	9
Anderson et al. (GUSTO-I)	1997	310	Obs	United States, Europe	STEMI	9
Barron et al. (NRMI-2)	2001	2990	Obs	United States	AMI	8
Gu et al	2010	91	Obs	Asia	STEMI	5
Prondzinsky et al. (IABP-SHOCK)	2010	40	RCT	Europe	AMI	7
Zeymer et al. (Euro Heart Survey PCI)	2012	653	Obs	Europe	STEMI or NSTEMI	8
Dziewierz et al. (EUROTRANSFER registry)	2014	51	Obs	Europe	STEMI	5
Brunner et al.	2019	42	Obs	Europe	AMI	5
Thiele et al. (IABP-SCHOCK II)	2012	598	RCT	Europe	AMI	7
Kim et al. (KAMIR)	2015	1214	Obs	Asia	AMI	8
ECMELLA vs. Impella						
Castro et al.	2020	27	Obs	Europe	ICMP(53.3%), DCM (26.7%)	6
ECMELLA vs. ECMO						
Pappalardo et al.	2016	63	Obs	Europe	STEMI (54%)	9
PATEL et al	2019	66	Obs	United States	STEMI (32%), NSTEMI (14%)	6
Tepper et al	2016	45	Obs	United States	AMI (26%), PCS (28%)	7
Schrage et al. (STOP-SHOCK)	2020	510	Obs	Europe	AMI (63%)	9
MOURAD et al	2018	16	Obs	Europe	AMI	5
AKANNI et al	2019	225	Obs	United States	AMI (25.78%), PCS (36.44%)	6
ECMO vs. Impella						
Wernly et al	2021	149	Obs	Europe	AMI (51%)	8
Lamarche et al	2010	61	Obs	Europe	ACS (39.3%)	8
Lemor et al.	2020	900	Obs	United States	AMI	7
Karami et al.	2020	128	Obs	Europe	AMI	8
Karatolios et al.	2020	166	Obs	Europe	AMI (86%)	8
ECMO plus IABP vs. PLVADs						
Kagawa et al.	2012	73	Obs	Asia	ACS, INCA, OHCA	9

### Primary outcomes

**Fig. 2:**
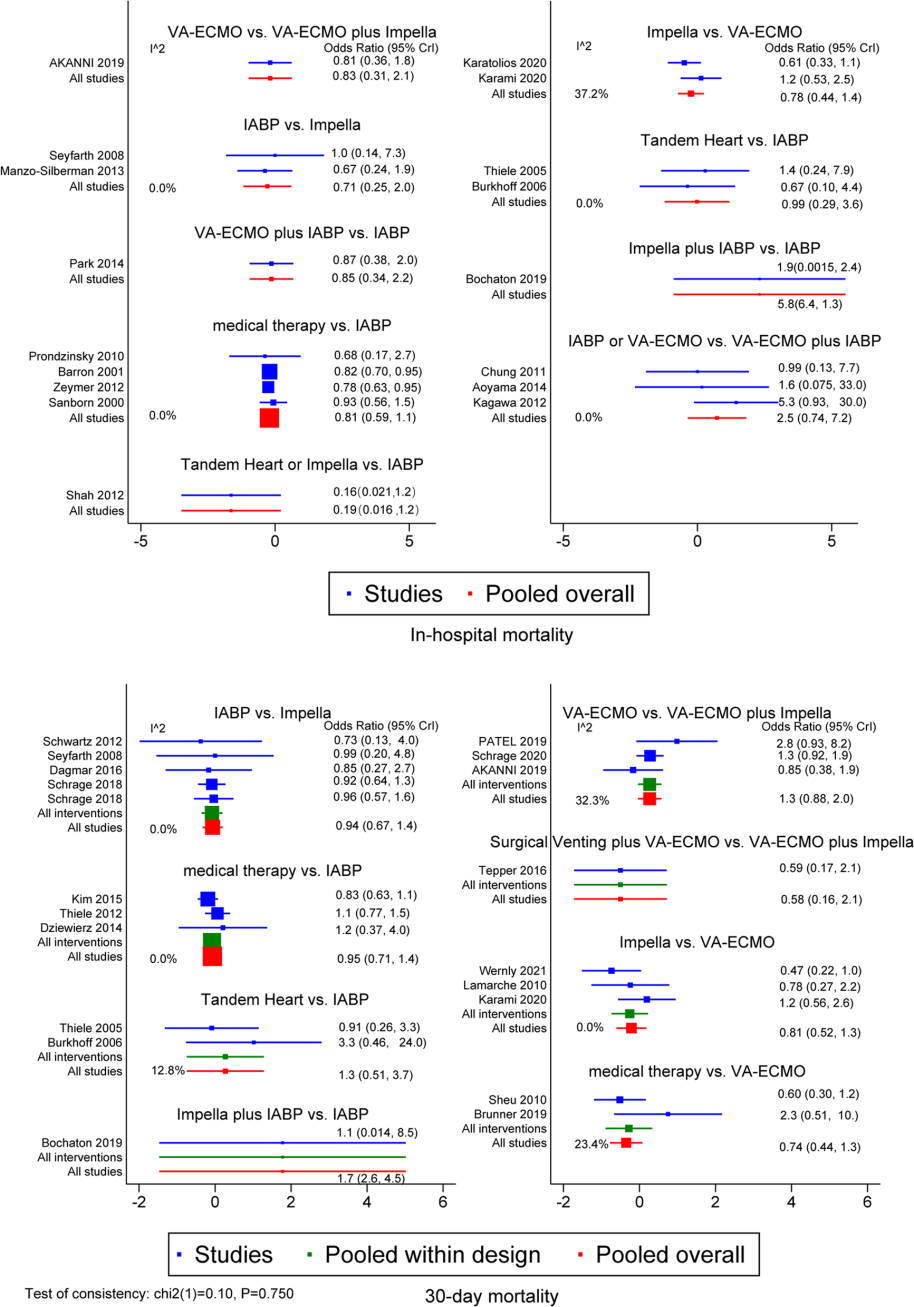
The forest plots of MCS for in-hospital mortality and 30-day mortality

Regarding in-hospital mortality, the results showed no significant differences between IABP and Impella, VA-ECMO plus IABP, Tandem Heart, and medical therapy (Fig. 2). According to the results of the SUCRA and cumulative ranking plots, the optimal ranking among the interventions was as follows: Tandem Heart or Impella, medical therapy, VA-ECMO plus IABP, PLVAD (Tandem Heart), IABP, Impella, VA-ECMO, IABP or VA-ECMO, VA-ECMO plus Impella, and Impella plus IABP (Additional file 1: Figures S2 and S3).

**Fig. 3:**
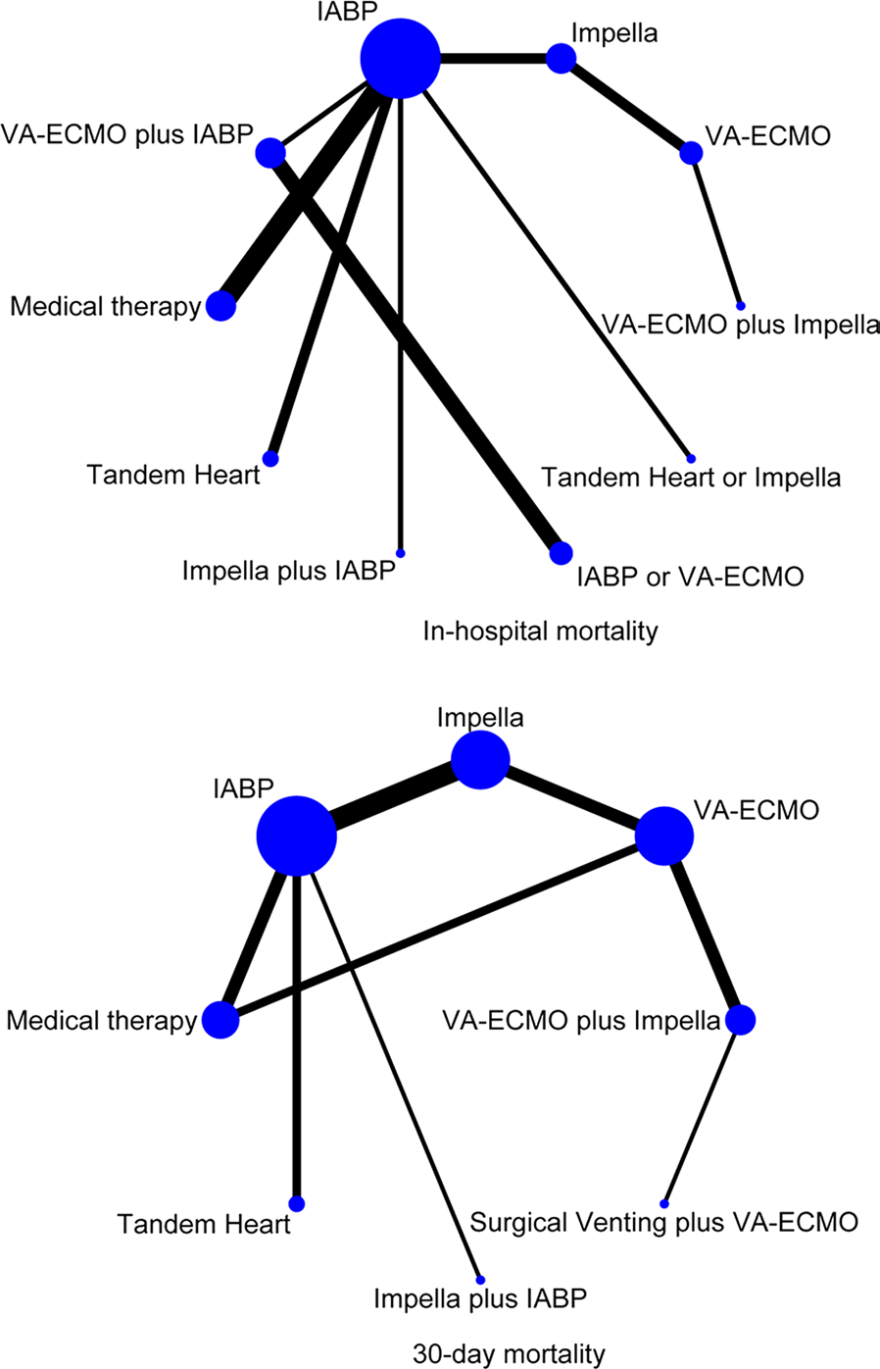
The network diagrams

Based on the in-hospital mortality and mortality within 30 days, we constructed two network diagrams (Fig. 3). The contribution of each study to the indirect comparison of interventions is shown in Additional file 1: Figure S4. Regarding 30-day mortality, the results showed no significant differences between VA-ECMO and VA-ECMO plus Impella, Impella, and medical therapy. In addition, no significant differences were noted between IABP, Tandem Heart, Impella, and medical therapy (Fig. 2). According to the results of the SUCRA and cumulative ranking plots, the optimal ranking among the interventions was as follows: surgical venting plus VA-ECMO, medical therapy, VA-ECMO plus Impella, IABP, Impella, Tandem Heart, VA-ECMO, and Impella plus IABP (Additional file 1: Figures S2 and S3).

### Heterogeneity and consistency

**Fig. 4:**
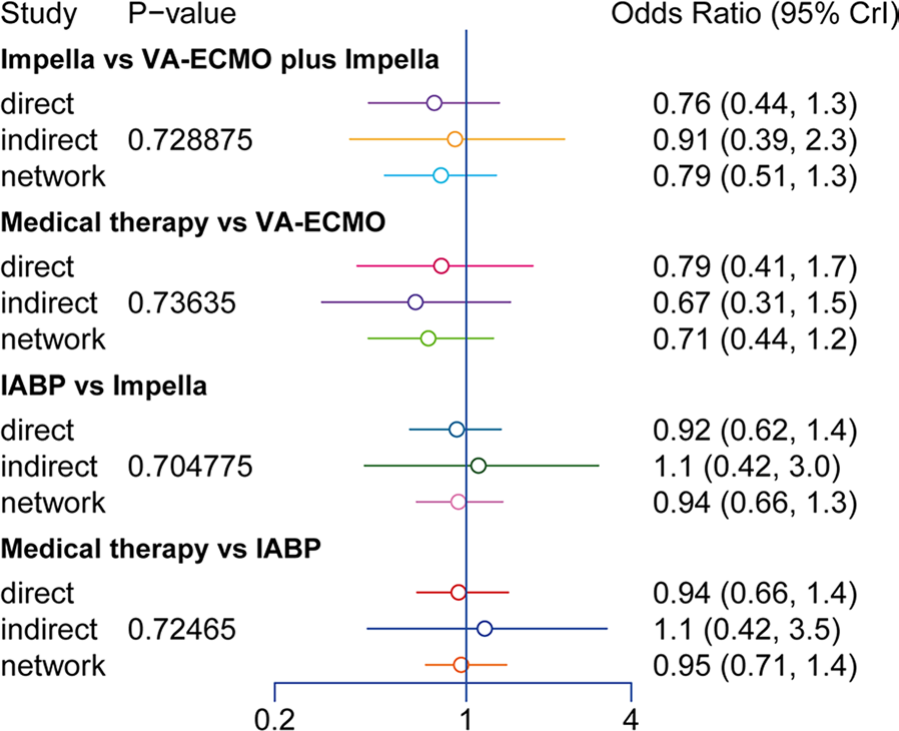
The consistency in direct and indirect comparisons of 30-day mortality

The forest plots showed that the heterogeneity of all results was low (Fig. 2). The results of the node-splitting approach showed relatively high consistency in direct and indirect comparisons (Fig. 4). *P* values were greater than 0.05. Density plots were used to judge the degree of convergence of the model. Additional file 1: Figure S5 demonstrates that the shape of the curve is close to a normal distribution. However, the intermediate value is far from “1”; the left side of the graph shows a better coincidence rate. In summary, the model had a good degree of fit.

### Bias detection and evidence for the NMA graded by the CINeMA system

**Fig. 5:**
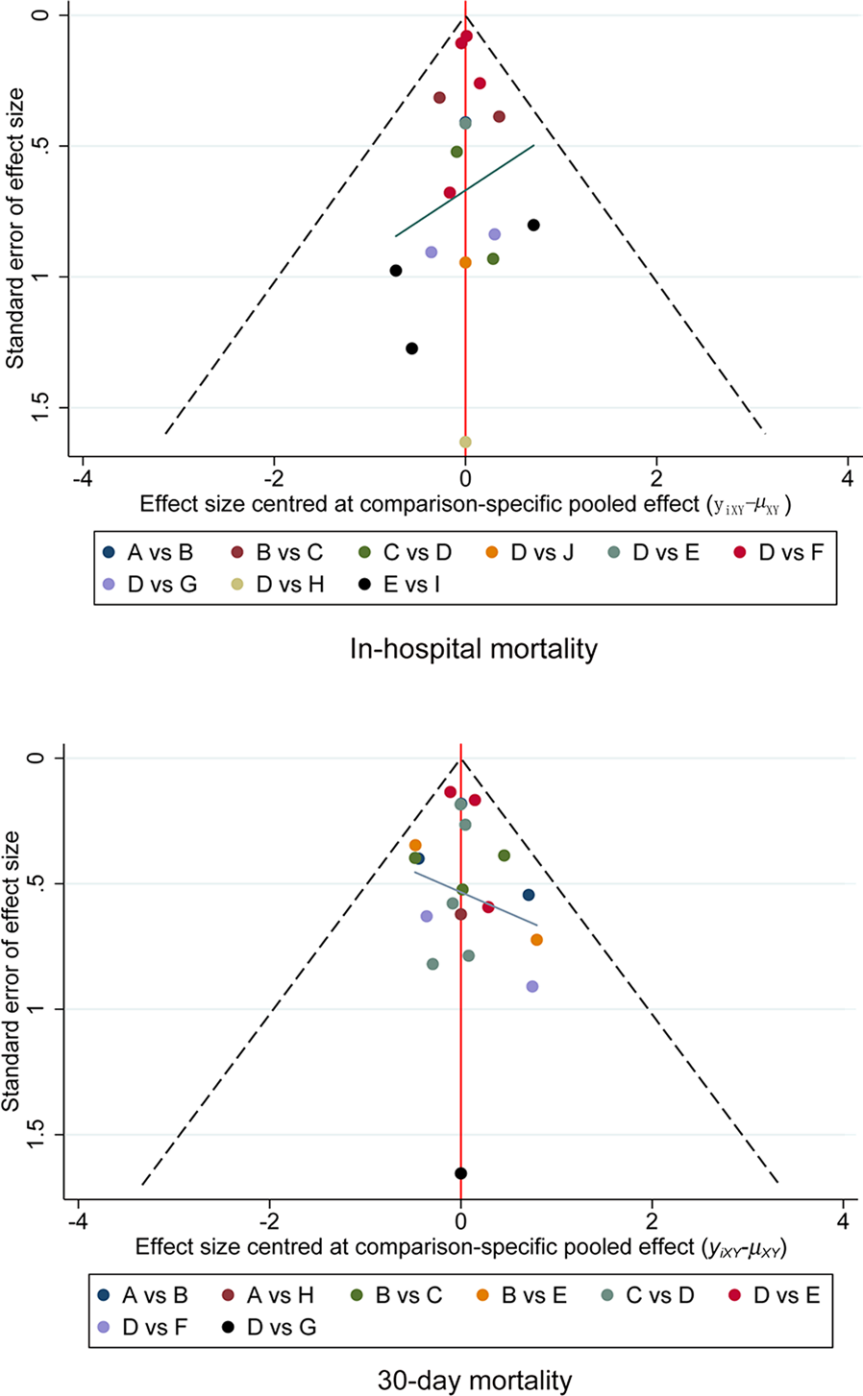
The funnel plot of all studies. (A) Venoarterial extracorporeal membrane oxygenation concomitant with Impella; (B) Venoarterial extracorporeal membrane oxygenation; (C) Impella; (D) Intra-aortic balloon pump; (E) Venoarterial extracorporeal membrane oxygenation plus Intra-aortic balloon pump; (F Medical therapy; (G) Tandem Heart; (H) Impella plus Intra-aortic balloon pump; (I) Venoarterial extracorporeal membrane oxygenation or Intra-aortic balloon pump; (J) Tandem Heart or Impella; (K) Surgical Venting

Regarding 30-day mortality, the funnel plot showed no significant bias in the included studies (Fig. 5). Given that this NMA includes observational trials and double-blind, randomized controlled trials, the evidence level of comparison between some interventions is low according to the CINeMA system.

## Discussion

Regarding 30-day mortality, the results of network comparison of VA-ECMO plus Impella versus VA ECMO, VA ECMO versus Impella, and IABP versus medical therapy showed high heterogeneity. Subsequently, sensitivity analysis was performed by omitting each study. Through sensitivity analysis, upon elimination of articles with a low-quality score, all results of the heterogeneity test showed low heterogeneity. Paired researchers reassessed the three articles with low-quality scores [21, 23, 56]. We believe that the reasons for the high heterogeneity may be related to the different aetiologies of CS and the different designs of the studies. For in-hospital mortality, the results of network comparison of VA-ECMO plus Impella versus VA ECMO, VA ECMO versus Impella, and IABP versus medical therapy also showed high heterogeneity. Subsequently, we also conducted a sensitivity analysis. Paired researchers reassessed the four articles with low-quality scores [23, 56, 58, 59]. The heterogeneity for all interventions was low following the exclusion of these four studies. Similarly, paired researchers discussed the reasons for the high heterogeneity. We agreed that the reason for the high heterogeneity may be the variations in the aetiology of CS and the study designs. After elimination of studies with low-quality scores, this NMA had a very favourable consistency, and the model had a comparatively favourable degree of conformity. In addition, most of the evidence levels of intervention comparison remained above medium. Regarding in-hospital mortality, the results of the SUCRA and cumulative ranking plots showed that Tandem Heart or Impella was superior to other interventions reducing in-hospital mortality. However, the studies of in-hospital mortality had a certain degree of publication bias. This notion reduced the level of evidence of Tandem Heart or Impella. In addition, compared with IABP plus Impella, IABP had a lower risk of in-hospital mortality (OR 5.89, 95% CI 1.33–6.4) and 30-day mortality (OR 1.78, 95% CI 2.6–4.56). After discussion among the researchers, the above results were considered to be less convincing. Only one study compared IABP plus Impella and IABP. Paired researchers reassessed the article with low-quality scores [60]. We cannot draw a conclusion from one study, which is unconvincing.

In this NMA, we included 39 clinical trials and evaluated the safety of various MCSs using the Bayesian method. For patients with CS, IABP is associated with the lower incidence of 30-day mortality than VA-ECMO and Impella.

VA-ECMO is a temporary mechanical circulatory support system that provides immediate and complete cardiopulmonary support in the event of CS and cardiac arrest [61].The centrifugal pump of VA-ECMO can propelup to 8 L/min of blood and promote cannula arterial return and venous drainage. A hollow fibre membrane oxygenator is spliced into the circuit, which not only provides blood oxygenation but also carbon dioxide (CO2) clearance via sweep gas flow. The latter function differentiates other MCS strategies, such as PLVADs and IABP [16]. Previously, strategies for LV unloading mainly included pulmonary vein or septal left atrial intubation, atrial septostomy, percutaneous mechanical circulatory support, transapical cannulation, or concomitant MCS devices, including IABP or PLVADs, such as Tandem-Heart [62–65]. However, many strategies require more difficult and invasive procedures with a considerable degree of correlation with serious complications [63]. Impella PLVAD (Abiomed, Danvers, MA) has been approved for use in the United States; in addition, it is also approved for the treatment of CS. The safety and effectiveness of VA-ECMO concomitant with Impella has been increasingly evaluated by several studies.

An increasing number of MCS devices have been developed for treating CS to enhance efficacy or to replace medical therapy to avoid potentially detrimental effects [66]. MCS devices can be classified based on the site of blood return, the sites from which blood is withdrawn from the body, their mechanism of action, and whether the devices provide carbon dioxide and oxygen gas exchange [66]. Devices include PLVADs, ECMO devices, percutaneous left atrial decompression devices, and aortic counterpulsation pumps. It should be noted that despite comparable effects on cardiac output and blood pressure, the effects of different forms of MCS on the heart and lung may be significantly different, specifically as determined by myocardial oxygen demand and pulmonary capillary wedge pressure (which is related to LV end-diastolic pressure) [67]. In addition, a scientific statement from the American Heart Association in 2017 noted little evidence for the selection of patients with CS who are suitable for MCS devices [68]. Therefore, in view of the feasibility and controversy of MCS in the treatment of CS patients, it is necessary to evaluate which type of MCS equipment has the superiority to better reduce mortality. MCS devices improve the systemic haemodynamics of CS patients by pumping blood from one vascular compartment to another, demonstrating the feasibility of MCS in the treatment of CS patients [67].

VA-ECMO has become a frequently used therapy for circulatory support during CS [69]. The clinical application of VA-ECMO has been widely accepted by doctors. However, VA-ECMO is still not easier to perform in the clinical setup with the improvement of peripheral cannulation. In addition, VA-ECMO might cause haemodynamic changes due to femoral artery retrograde flow, which can increase cardiac afterload and may also cause an increase in pulmonary capillary wedge pressure and left ventricular end diastolic pressure(LVEDP), which will eventually lead to the occurrence of pulmonary oedema and an increase in myocardial oxygen consumption [70, 71]. Furthermore, the associated phenomenon of LV distention cannot be ignored. LV distention is typically associated with ventricular arrhythmias and stasis of blood in the LV. Therefore, during the use of VA-ECMO, the use of a second MCS device offers great potential theoretical advantages, which play an important role in reducing myocardial oxygen consumption, pulmonary oedema, and LV distention [70, 72]. For traditional LV unloading strategies, in addition to surgical venting, IABP has always been considered a mainstream intervention. However, sufficient evidence is not available to demonstrate the capacity of IABP to reduce the occurrence of vascular adverse events. More researchers believe that the effectiveness of IABP in CS is reduced because the haemodynamic support produced by IABP is closely related to the cardiac output produced by the ventricle itself [73–75]. With the advancement of Impella technology, an Impella rotary pump can generate 2.5–3.5 L of blood flow, which plays a considerable role in improving coronary perfusion, and can greatly improve haemodynamic endpoints, thereby compensating for the shortcomings of IABP [51, 76]. Although Impella can significantly improve coronary perfusion, there is still a risk of haemolysis, which is a common problem noted among pump devices [77]. Therefore, the VA-ECMO plus Impella intervention strategy can be more beneficial in the treatment of CS patients as it can significantly reduce the central venous pressure compared with VA-ECMO alone [31, 38]. Related studies have shown that among AMI patients complicated by CS, the use of PLVAD is associated with a significantly higher risk of in-hospital mortality and haemorrhage compared with IABP [68]. However, it can not be ignored that despite the early use of IABP, the prognosis of patients with CS remains poor [78].

Regarding the use of Impella, haemolysis is a known common complication associated with acute renal failure and increased demand for blood transfusions [77]. In addition, bleeding is also a common complication of the use of MCS equipment during CS, which is related to vascular damage caused by arterial and venous cannulation [79]. When using VA-ECMO and Impella, it is necessary to administer a sufficient dose of anticoagulants to prevent thrombosis. This process enhances the risk of bleeding [80]. Acute renal failure is also a treatment challenge faced by clinicians. However, prolonging survival is considered to be the ultimate goal of CS management. Therefore, it is of great significance to evaluate the safety of various MCSs for CS patients. The various aetiologies of CS included in the NMA may have a certain degree of influence on the results of this study. Therefore, it is necessary to discuss the baseline data of this study. The aetiologies of CS in this NMA include unstable angina (UA), acute myocardial infarction (AMI), in-of-hospital cardiac arrest (INCA), out-of-hospital cardiac arrest (OHCA), ischaemic cardiomyopathy (ICMP), and dilative cardiomyopathy (DCM). However, after the exclusion of studies with low-quality scores, the heterogeneity, consistency, and convergence of the model had good results, which may be related to the analysis of the sole event of death in this NMA. However, MCS equipment is adopted for the treatment of CS patients, and mortality data provide a very important reference for clinicians to specify the diagnosis and treatment plans. This study compared the pros and cons of various MCS interventions. In addition, in this NMA, some interventions have been included in a small number of clinical trials, resulting in a small sample size for those interventions. However, as the applications of MCS are gradually recognized by clinicians, further clinical studies on MCS devices will emerge, to assess their clinical safety.

The present study is the first network meta-analysis of various MCS interventions, and it explores the best intervention strategy for the treatment of CS. In addition, the study makes an indirect comparison between interventions that were not included in clinical research. In addition, 39 articles and 10,985 patients were included in this NMA, which makes our results more credible. However, the aetiologies of CS that are not fully controlled may represent the shortcomings of our research.

## Conclusions

IABP is recommended to reduce 30-day mortality in CS patients.

### Review registration

PROSPERO, CRD42021282526

## Supplementary Information

**Additional file 1.** Supplemental Figures.

## Data Availability

All data generated or analyzed during this study are included in this manuscript and its additional files.
