# Supplementary material for: Mortality in cardiogenic shock patients receiving mechanical circulatory support: a network meta-analysis
Source: BMC Cardiovasc Disord. 2022 Feb 13;22:48. doi: 10.1186/s12872-022-02493-0 (PMC8842943; doi:10.1186/s12872-022-02493-0)
Supplement: Supplementary file 1 — Additional file 1. Supplemental Figures. [file 12872_2022_2493_MOESM1_ESM.docx]

Supplemental Data


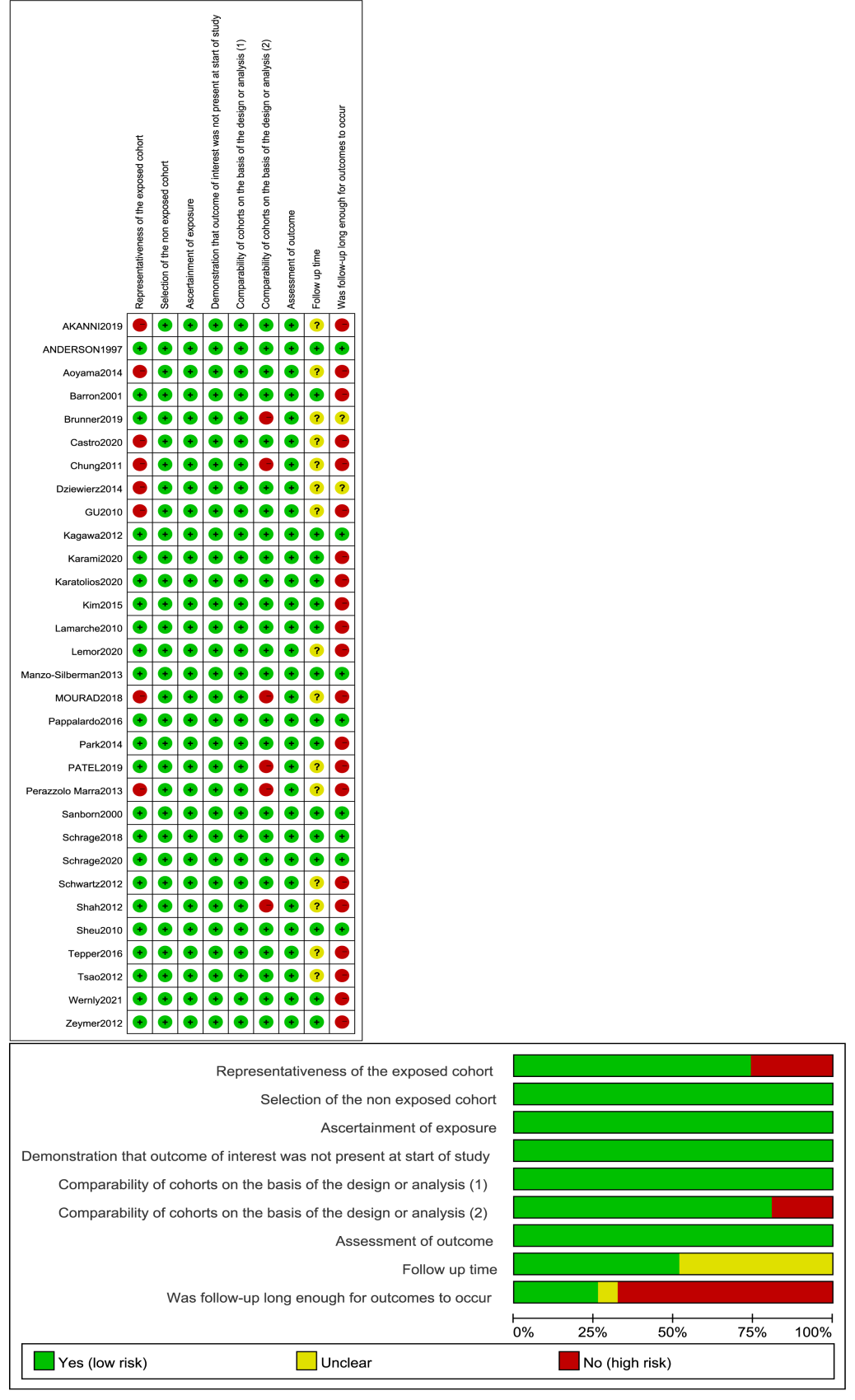


Supplemental Figure 1. The quality assessment of observational trials


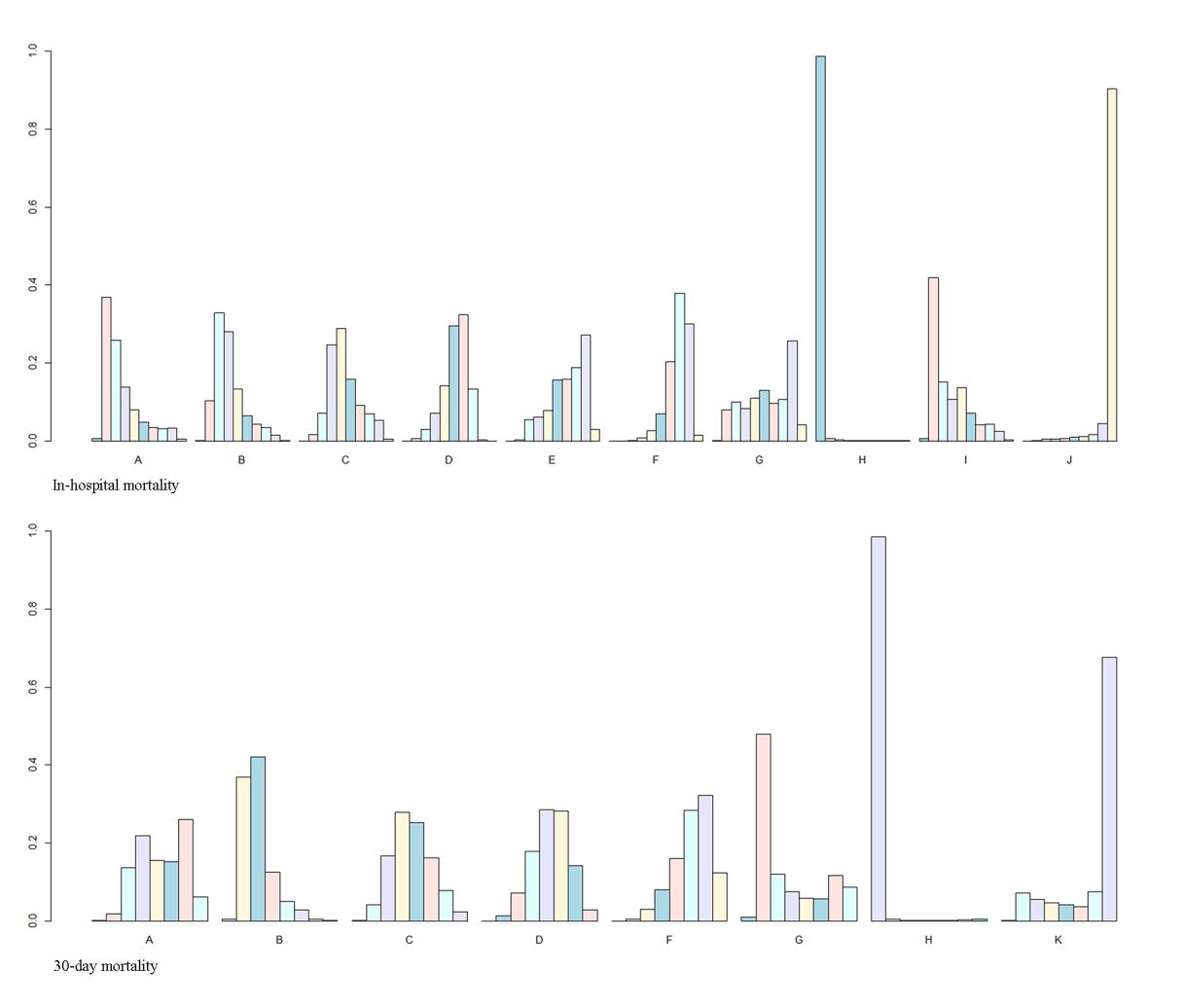


Supplemental Figure 2. The cumulative ranking plots interventions of in-hospital and 30-day mortality. A, Venoarterial extracorporeal membrane oxygenation concomitant with Impella; B, Venoarterial extracorporeal membrane oxygenation; C, Impella; D, Intra-aortic balloon pump; E, Venoarterial extracorporeal membrane oxygenation plus Intra-aortic balloon pump; F, Medical therapy; G, Tandem Heart; H, Impella plus Intra-aortic balloon pump; I, Venoarterial extracorporeal membrane oxygenation or Intra-aortic balloon pump; J, Tandem Heart or Impella; K, Surgical Venting.


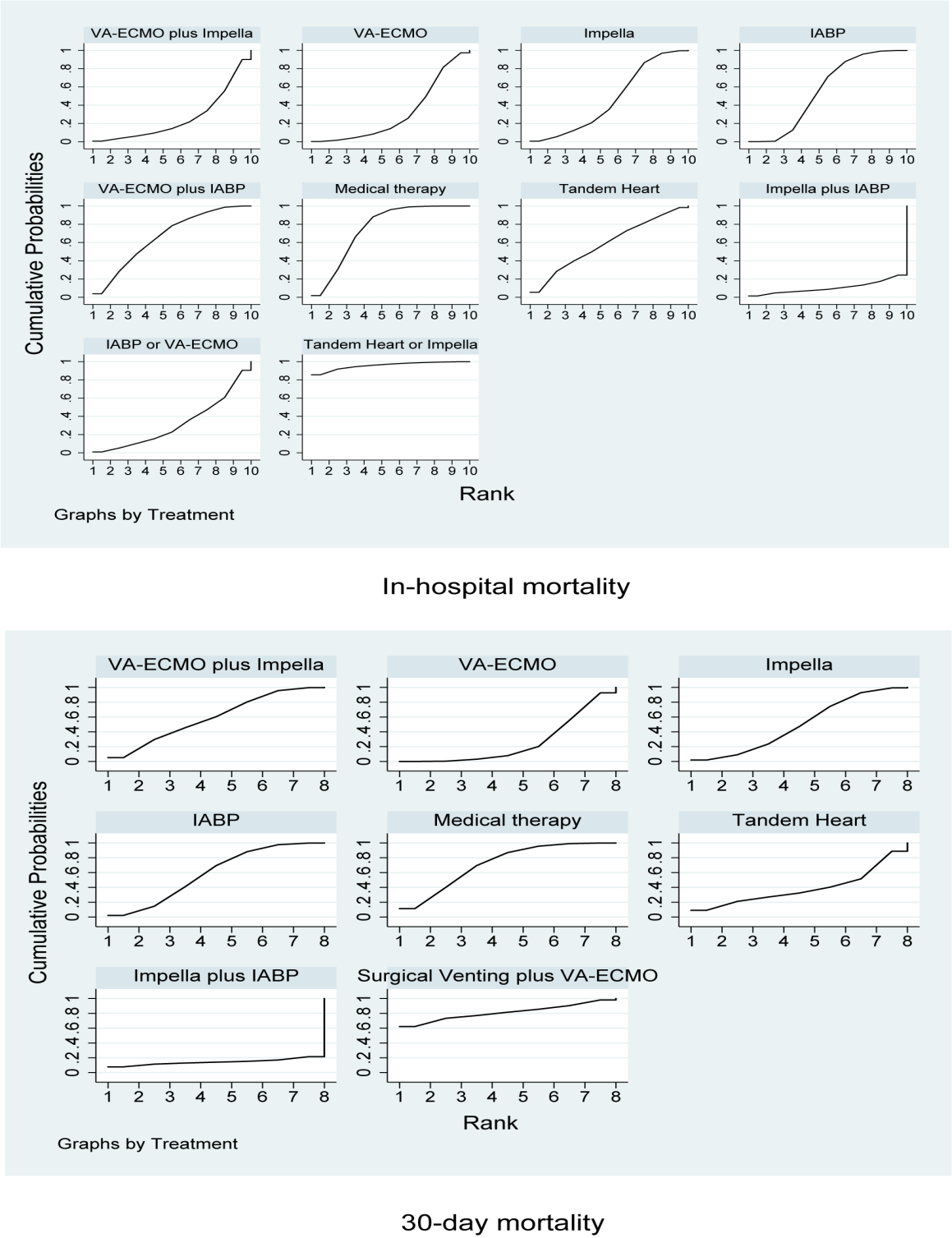


Supplemental Figure 3. The SUCRA of in-hospital mortality and 30-day mortality.


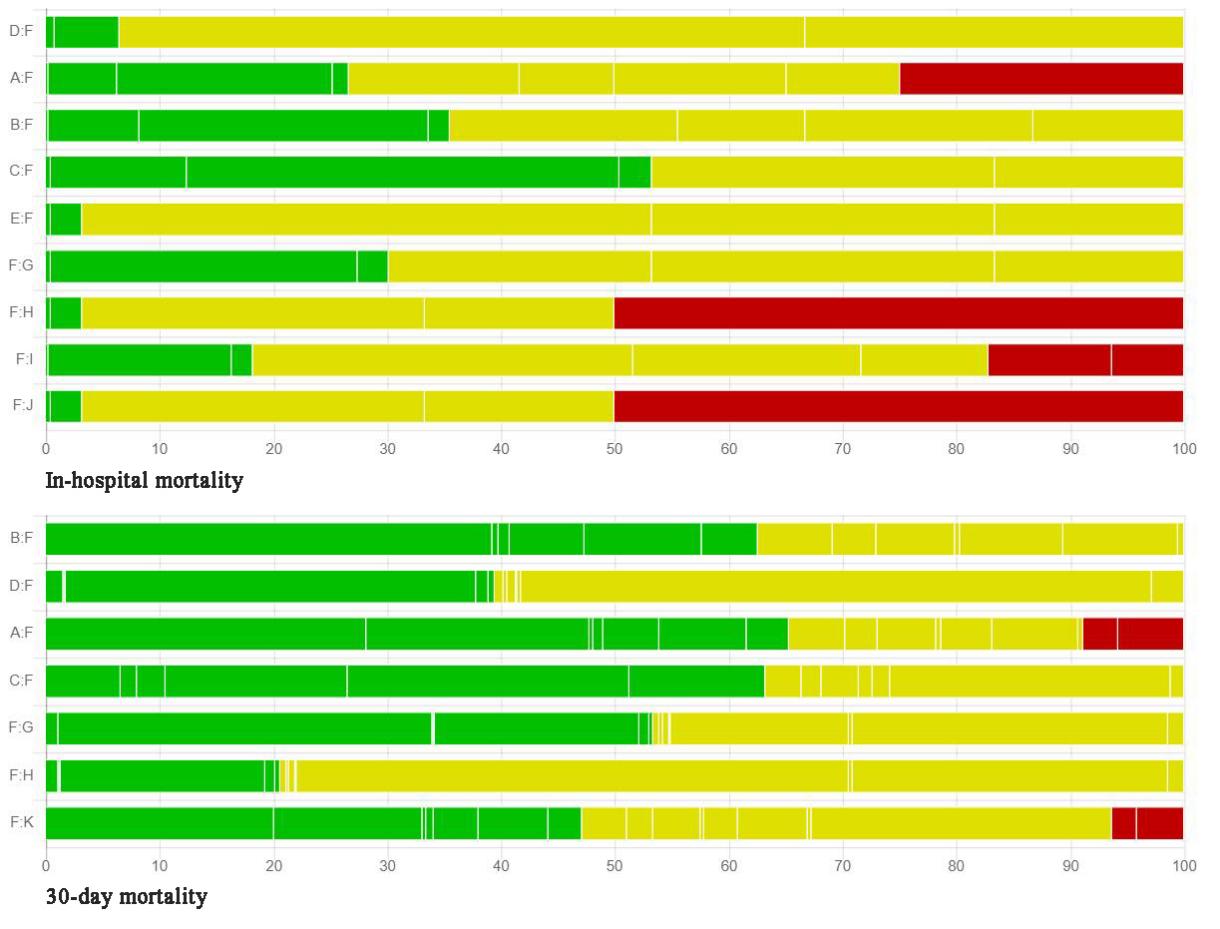


Supplemental Figure 4. The contribution of each study to the indirect comparison of interventions. A, Venoarterial extracorporeal membrane oxygenation concomitant with Impella; B, Venoarterial extracorporeal membrane oxygenation; C, Impella; D, Intra-aortic balloon pump; E, Venoarterial extracorporeal membrane oxygenation plus Intra-aortic balloon pump; F, Medical therapy; G, Tandem Heart; H, Impella plus Intra-aortic balloon pump; I, Venoarterial extracorporeal membrane oxygenation or Intra-aortic balloon pump; J, Tandem Heart or Impella; K, Surgical Venting.


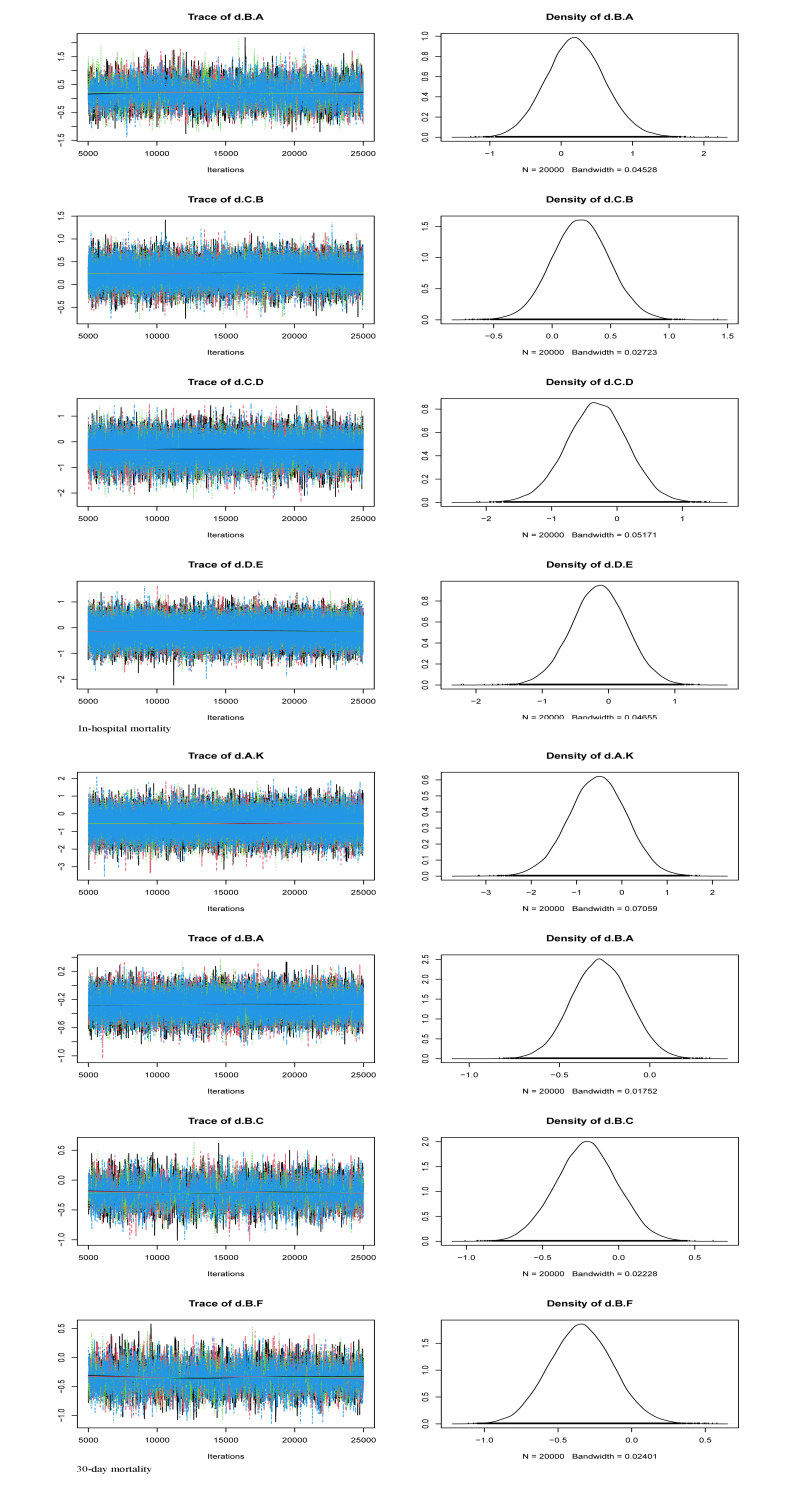


Supplemental Figure 5. The density plots of in-hospital and 30-day mortality. A, Venoarterial extracorporeal membrane oxygenation concomitant with Impella; B, Venoarterial extracorporeal membrane oxygenation; C, Impella; D, Intra-aortic balloon pump; E, Venoarterial extracorporeal membrane oxygenation plus Intra-aortic balloon pump; F, Medical therapy; K, Surgical Venting.
